# Late arrhythmia recurrence after atrial fibrillation ablation: incidence, mechanisms and clinical implications

**DOI:** 10.1007/s00399-021-00836-6

**Published:** 2022-01-10

**Authors:** Nico Erhard, Andreas Metzner, Thomas Fink

**Affiliations:** 1 Department of Electrophysiology, German Heart Centre Munich, Lazarettstr. 36, 80636 Munich, Germany; 2 Department of Cardiac Electrophysiology, University Heart Center, University Hospital Hamburg Eppendorf, Hamburg, Germany; 3 Clinic for Electrophysiology, Herz- und Diabeteszentrum NRW, Ruhr-Universität Bochum, Bad Oeynhausen, Germany

**Keywords:** Atrial fibrillation, Catheter ablation, Pulmonary vein isolation, Late recurrences, Atrial fibrosis, Arrhythmia recurrence, Vorhofflimmern, Katheterablation, Pulmonalvenenisolation, Spätrezidive, Atriale Fibrose, ??

## Abstract

**Background and objectives:**

Catheter ablation of atrial fibrillation (AF) has become a well-established and widely used therapy, with pulmonary vein isolation (PVI) being the key modality of ablation. However, arrhythmia recurrences after PVI are common, with a relevant number of patients undergoing repeat ablation. Arrhythmia recurrence after PVI may vary regarding time point and mode of recurrence. While early arrhythmia recurrences of AF after PVI are mostly found to be the product of electrical reconnection of the pulmonary veins, the exact mechanisms of very late arrhythmia recurrence, occurring later than 12 months after successful PVI, remain unclear. This review provides an overview on the current evidence on time point and mechanisms of arrhythmia recurrence after PVI focussing on late arrhythmia recurrence.

**Recent findings:**

The incidence of late arrhythmia recurrence after PVI can lie at a rate of up to 30% according to long-term follow-up studies. Mechanisms of recurrence include electrical reconnection of previously isolated pulmonary veins and development of atrial fibrosis. The use of cryoballoon ablation is likely to be more effective in reducing late arrhythmia recurrences compared to radiofrequency ablation. Novel scores such as the MB-LATER score or the APPLE score may become useful tools in predicting arrhythmia recurrence after PVI.

**Results and conclusion:**

Late arrhythmia recurrence after PVI is common and leads to a relevant impairment of long-term success. Relevant data are currently limited and exact mechanisms of arrhythmia recurrence remain unclear. Further studies are needed to elucidate pathogenetic mechanisms of late arrhythmia recurrence after PVI in order to improve treatment strategies.

## Abstract (de)

**Hintergrund und Ziel der Arbeit:**

Die Katheterablation von Vorhofflimmern (VHF) hat sich zu einer etablierten und weit verbreiteten Therapie entwickelt, wobei die Pulmonalvenenisolation (PVI) die wichtigste Ablationsmethode darstellt. Dennoch sind Arrhythmierezidive häufig und eine relevante Anzahl von Patienten benötigt wiederholte Ablationen. Rezidive nach PVI können hinsichtlich des Rezidivzeitpunkts und unterliegenden Mechanismus variieren. Während frühe VHF-Rezidive nach PVI meist auf die elektrische Rekonnektion der Pulmonalvenen zurückzuführen sind, bleiben die genauen Mechanismen sehr später Arrhythmierezidive, die mehr als 12 Monate nach erfolgreicher PVI auftreten, unklar. Die vorliegende Übersichtsarbeit fasst die aktuelle Studienlage zu Zeitpunkt und Mechanismen von Arrhythmierezidiven nach PVI zusammen. Der Schwerpunkt liegt dabei auf Spätrezidiven.

**Ergebnisse:**

Die Inzidenz von späten Arrhythmierezidiven nach PVI kann laut Langzeitstudien bis zu 30 % betragen. Zu den unterliegenden Mechanismen gehören die elektrische Rekonnektion zuvor isolierter Pulmonalvenen und die Entwicklung atrialer Fibrosen. Die Kryoballonablation verringert späte Arrhythmierezidive potenziell wirksamer als die Radiofrequenzablation. Kürzlich entwickelte Scores wie der MB-LATER-Score oder der APPLE-Score könnten sich als nützliche Instrumente für die Vorhersage eines Arrhythmierezidivs nach PVI erweisen.

**Schlussfolgerung:**

Das späte Wiederauftreten von Arrhythmien nach PVI ist ein häufiges Phänomen, das den langfristigen Therapieerfolg erheblich beeinträchtigt. Derzeit liegen nur wenige Daten zu diesem Thema vor und die genauen Mechanismen später Arrhythmierezidive sind unklar. Weitere Studien sind erforderlich, um die unterliegenden pathogenetischen Mechanismen aufzuklären und somit Behandlungsstrategien verbessern zu können.

## Introduction

Catheter ablation has become an accepted first line therapy for paroxysmal atrial fibrillation (PAF), with pulmonary vein isolation (PVI) being the key element of ablation strategies [1, 2]. Catheter ablation of AF has been proven to be superior to antiarrhythmic drug (AAD) therapy regarding efficacy and improvement of survival in patients with heart failure [3, 4].

However, early recurrence of AF (ERAF) within the first 3 months after PVI using radiofrequency (RF) energy is a clinically relevant problem with up to 35% of patients needing repeat ablation procedures [5, 6]. Early arrhythmia recurrence within the first 3 months after PVI is mostly associated with early inflammation, incomplete scar formation and pulmonary vein reconnection [5, 7, 8].

In contrast, data on arrhythmia recurrence occurring at later than 1 year, or even after multiple years after PVI, are limited and the exact mechanisms behind late arrhythmia recurrence after PVI have not been clearly identified to date [9, 10].

The present article provides the current scientific view on the mechanisms and incidence of arrhythmia recurrence after PVI focussing on late arrhythmia recurrence.

## Efficacy of AF ablation

PVI has become a routine treatment procedure in recent decades especially for patients with symptomatic PAF [4, 5].

A systematic meta-analysis from 2009 investigating 31 different studies found a single procedure success rate of 57% and a multiple procedure success rate of 71% in patients receiving radiofrequency ablation (RFA) without any anti-arrhythmic drug therapy [11].

A 5-year follow-up published in 2010, which investigated the outcome of PVI using RFA in patients with PAF, found a 46.6% success rate in patients undergoing only one ablation procedure. Success was defined as a stable sinus rhythm for 5 years after the procedure, with success rates increasing to 73.9% when additional procedures were performed. A total of 72.1% of recurrences took place within the first 12 months after the initial ablation procedure [12].

As most arrhythmia recurrences take place within the first 3 months after the initial procedure, this time period is seen as a so-called “blanking period”, in which ablation is not recommended, since up to 50% of patients can become arrhythmia-free in the long run [5].

Ablation therapy, however, is less successful in patients suffering from longstanding persistent AF, with a 20.3% success rate after a single procedure and a 45% success rate after multiple RFA procedures according to a 5-year follow up conducted in 2012 [13].

The STAR AF II Trial published in 2015 investigated different ablation strategies when treating longstanding persistent AF, addressing the higher recurrence rates when treating persistent AF compared to PAF. However, no significant difference regarding arrhythmia-free survival was found between PVI only compared to linear lesions ablation only or ablation of complex fractionated electrograms [14].

In comparison to RF energy the use of cryoballoon technology (CB) as an ablation method for PAF has shown significantly higher successful procedural outcomes with 60.3–72.8% of patients being free from arrhythmia after a single procedure has been performed [15–17]. Another study, however, identified that the rate of early AF recurrences still lies at about 50% after a single procedure when using CB technology [18].

## Early arrhythmia recurrence

Repeat ablation procedures are mostly performed after a blanking period since early arrhythmia recurrence after PVI is often associated with inflammation and inhomogeneous scar tissue formation after ablation [5]. A blanking period of 3 months is common clinical practice today [19]. AF recurrence within that timeframe is referred to as ERAF and, despite being common, is considered a risk factor for late AF recurrences with long-term clinical impact [1, 5, 10].

Especially patients suffering from AF recurrences in the third months of the blanking period have an up to 90% chance of being symptomatic again after 1 year [19].

An arrhythmia-free period of 1 month after the ablation, on the other hand, does not necessarily guarantee long-term arrhythmia-free survival [20].

The effect of prevention of early recurrences on long-term arrhythmia-free survival has not been evaluated as yet [6, 21].

## Long-term success of catheter ablation

It is well known that untreated PAF often progresses to persistent AF and ultimately results in acceptance of permanent AF. Therefore, early treatment of PAF is critical to improve long-term outcome [22, 23].

In the majority of patients suffering from PAF catheter ablation results in stable sinus rhythm as shown in long-term follow-up studies [5, 20]. However, most patients need multiple ablation procedures to restore durable sinus rhythm [24, 25]. According to one meta-analysis study, patients suffering from PAF undergoing multiple ablation procedures using RF energy have a long-term success rate of about 80%, whilst success rates for patients undergoing only one ablation procedure is around 53% [24]. Recent data suggest there are several reasons for this significant difference, with pulmonary vein reconnection probably being the most prominent one [26]. Long-term success rates are significantly lower in patients, with persistent AF resulting in only a 20–45% rate of 5‑year arrhythmia-free survival after multiple RFA [13, 27]. The ideal ablation strategy for patients with persistent AF has not yet been identified [21, 27].

## Incidence of very late arrhythmia recurrence

Very late arrhythmia recurrences after PVI are commonly defined as recurrent AF occurring 12 months or later after the last procedure [5]. Most studies agree that pulmonary vein reconnection and the development of non PV triggers are the main mechanism behind these recurrences [26, 28].

A 2008 study from New York providing 28-month follow-up post RF PVI in patients suffering from PAF discovered that late recurrence of AF occurs in 8.7% of patients. The study also concluded that there was a significantly higher incidence of hypertension and dyslipidaemia in these patients [26].

Weerasooriya et al. described an arrhythmia-free survival rate of 63% in PAF patients 5 years after the last RFA, and noted a gradual increase in recurrence rates over time [29]. Another study showed that there was an increase from 27 to 39% in recurrence rates (1 and 2.5 years after ablation) in patients suffering from PAF. The recurrence rates were even higher (from 37 to 57%) in patients initially suffering from persistent AF [30].

This thesis is also underlined in the 2017 expert consensus statement on AF ablation, which states that an increase in follow-up duration also increases the likelihood of arrhythmia recurrences [5].

A study from 2016 investigated late recurrences after CB PVI and described a late arrhythmia recurrence rate of 69% in patients who already suffered from ERAF. On the other hand, there was an only 9.3% late recurrence rate in patients not having previously suffered from ERAF. This underlines the fact that the likelihood of late arrhythmia recurrence is drastically increased when ERAF had occurred [31]. A summary of all reviewed publications can be found in Table 1.

## Mechanisms of early and late arrhythmia recurrence after ablation

One of the identified main causes of atrial fibrillation recurrences after PVI is electrical reconnection within the pulmonary veins [24, 32, 33]. Early recurrence within the first month of the procedure lies at around 27 and 35% [6, 34]. A recent study from Pennsylvania has found reconnection of previously isolated pulmonary vein segments in 97% of patients and identified these electrical reconnections as a trigger of recurrent atrial fibrillation [35]. Other studies additionally described that the majority of triggers of recurrent AF after PVI originated from electrically reconnected areas within the pulmonary veins rather than from newly formed trigger points [7]. The rate of pulmonary vein reconduction is 80% within the first 4 month after RFA [33]. Patients suffering from ERAF are considered to be more likely to suffer from AF in the long run [19].

Although pulmonary vein reconnection is the major issue concerning early recurrences of AF after PVI, experts believe that individual risk factors may be of importance [24, 36]. A recent study from Hamburg identified body mass index (BMI) as a main risk factor for developing AF, and a study from 2013 investigating the predicting factors of late AF recurrence identified obesity, metabolic syndrome, procedural failure and ERAF as clear independent risk factors for late recurrence [37, 38].

One study identified that patients with risk factors such as high BMI who underwent risk factor management (RFM) after their RFA had better long-term outcomes regarding AF recurrences compared to patients not receiving RFA [39].

Especially a high amount of pericardial fat can result in a less successful long-term RFA outcome in all subtypes of AF and can also lead to a higher symptom burden for patients [40, 41]. The correlation between AF recurrences and the amount of pericardial fat may be stronger than mere BMI [40].

A study from 2015 showed that the CHADS2 or CHA2DS2-VASc score, heart failure and stroke were effective in predicting the procedural outcome after RFA over 5 years for all AF subtypes [42].

Patients suffering from AF recurrence after PVI are also likely to have hyperlipidaemia, hypertension or obstructive sleep apnoea (OSA) [26, 39, 43]. OSA was identified to be the most accurate predictor of AF recurrence with only 41% arrhythmia-free patients 4–7 months after PVI compared to 63% arrhythmia-free patients without OSA [43, 44]. Atrial remodelling in patients with OSA may be causative for higher recurrence rates [45]. Therefore, treating OSA in AF patients with continuous positive airway pressure (CPAP) ventilation reduces their risk of arrhythmia recurrence after PVI [46, 47].

Furthermore, AF has been associated with genetic predispositions [48, 49]. Several Studies have identified a potential genetic predisposition regarding recurrences of AF after PVI [50, 51]. For example, patients with polymorphisms on chromosome 4q25 were at higher risk for generally developing AF as well as for developing early and late arrhythmia recurrence after PVI [52, 53].

## Influence of the ablation approach on AF recurrences

In addition to predispositions and individual risk factors the ablation approach can also influence short- and long-term outcome [5]. For example, experts believed that using the technique of an atrial line in the repeat RF PVI might decrease the likelihood of developing PAF recurrences. A study from Munich, Germany, however, has proved this theory wrong, showing instead that there is no significant difference regarding AF recurrences when using an atrial line + PVI versus using a standard RF PVI [54].

As PV reconnection is a major cause of ERAF, detecting reconnected trigger points as early as possible can be very effective in improving procedural success [24]. This rate of early reconnection of the PV can be reduced by incorporating a waiting period of 1 h after the initial procedure. Implementing this waiting period into clinical practice could help detect early reconnections, thus reducing the rate of AF recurrences [55, 56].

The increasingly used CB for PVI might also influence the rate of early and late AF recurrences [5]. The fire and ice trial has already identified CB-based PVI as equally effective compared to RF-based PVI in patients with PAF [16]. According to a 2014 study up to 50% of patients with PAF receiving CB ablation suffered from ERAF. In the study early reablation was strongly associated with a lower incidence of late AF recurrences, with only 3.3.% of patients developing a late recurrence after a repeat ablation and 55.6% developing recurrences without receiving reablation [18].

The more recently developed cryoballoon 2 has shown even better procedural outcomes, with 83.6% of PAF patients being free from AF recurrences after a single PVI was performed [57, 58]. Additionally, the rate of PV reconnection appears to be significantly lower when using CB compared to RFA [59].

## Prediction of arrhythmia recurrences

For better prediction of arrhythmia recurrence, predictive scores have become an important tool. A new score from 2017 is the APPLE Score. It includes several factors such as age > 65 years, persistent AF, impaired estimated glomerular filtration rate (< 60 ml/min/1.73 m2), left atrium diameter ≥ 43 mm and an ejection fraction < 50%, with each factor counting for one point. A study from Leipzig, Germany, has shown that this scoring system is a superior predictor of AF recurrences after repeat ablation compared to scores such as CHADS2 and CHA2DS2-VASc [60]. Another promising new score is the MB-LATER Score (male, bundle branch block, left atrium ≥ 47 mm, type of AF [paroxysmal, persistent or long-standing persistent AF], and ERAF). One study that compared different scores focused entirely on predicting AF recurrences after PVI and used the area under the curve (AUC) as an indicator for the predictive value of the scores. The study showed that the MB-LATER Score provided a more accurate prediction of very late AF recurrence (AUC 0.782) than other scoring systems (including the APPLE score [AUC 0.716]) [61].

A more recent study from Leipzig, however, disagreed with these findings, stating that while other scoring systems are useful, early recurrence of AF remains the clearest and most precise indicator of late arrhythmia recurrence. The authors concluded that an accurate AF recurrence prediction score that can be fully implemented into clinical practice has not yet been found [62].

## Conclusion

PVI has become an accepted treatment for PAF. However, recurrences especially within the first 3 months post ablation may occur, with most patients needing more than one ablation to maintain stable sinus rhythm. The mechanism of electrical reconnection of the pulmonary veins is the main reason for early and late AF recurrences. However, individual risk factors like BMI and obesity definitely may affect likelihood of arrhythmia recurrence also after PVI. CB-based PVI might be beneficial in order to reduce early and late arrhythmia recurrence rates after PVI. However, long-term follow-up studies focusing on late AF recurrences after CB ablation are limited.

Several scoring systems, such as the MB LATER Score, have been developed to predict AF recurrences but need further evaluation in larger studies before routine clinical use. All in all, studies investigating late arrhythmia recurrences after PVI are sparse and more studies need to be conducted in future for a better understanding and effective management.

**Table 1:** Summary of publications reviewed

Reference	Number of patients	Underlying arrhythmia	Follow-up (month)	Recurrence rate (%)
[26]	350	PAF	28 ± 12	8,7
[30]	428	PAF	36 ± 24	29
[30]	346	PeAF	36 ± 24	37
[12]	161	PAF	60	20,5
[29]	100	PAF/PeAF	60	37
[13]	202	LAF	56 ± 8	53,3
[20]	125	PAF	36	15
[20]	79	PeAF	36	25

*PAF* paroxysmal atrial fibrillation; *PeAF* persistent atrial fibrillation; *LAF* longstanding persistent atrial fibrillation

## Declarations

For this article no studies with human participants or animals were performed by any of the authors. All studies performed were in accordance with the ethical standards indicated in each case.
